# Plant Glycan Metabolism by Bifidobacteria

**DOI:** 10.3389/fmicb.2021.609418

**Published:** 2021-02-04

**Authors:** Sandra M. Kelly, Jose Munoz-Munoz, Douwe van Sinderen

**Affiliations:** ^1^School of Microbiology and APC Microbiome Ireland, University College Cork, Cork, Ireland; ^2^Microbial Enzymology Group, Department of Applied Sciences, Northumbria University, Newcastle upon Tyne, United Kingdom

**Keywords:** bifidobacteria, plant glycans, plant oligosaccharides, fiber, glycosyl hydrolase, CAZy enzymes, carbohydrate metabolism

## Abstract

Members of the genus *Bifidobacterium*, of which the majority have been isolated as gut commensals, are Gram-positive, non-motile, saccharolytic, non-sporulating, anaerobic bacteria. Many bifidobacterial strains are considered probiotic and therefore are thought to bestow health benefits upon their host. Bifidobacteria are highly abundant among the gut microbiota of healthy, full term, breast-fed infants, yet the relative average abundance of bifidobacteria tends to decrease as the human host ages. Because of the inverse correlation between bifidobacterial abundance/prevalence and health, there has been an increasing interest in maintaining, increasing or restoring bifidobacterial populations in the infant, adult and elderly gut. In order to colonize and persist in the gastrointestinal environment, bifidobacteria must be able to metabolise complex dietary and/or host-derived carbohydrates, and be resistant to various environmental challenges of the gut. This is not only important for the autochthonous bifidobacterial species colonising the gut, but also for allochthonous bifidobacteria provided as probiotic supplements in functional foods. For example, *Bifidobacterium longum* subsp. *longum* is a taxon associated with the metabolism of plant-derived poly/oligosaccharides in the adult diet, being capable of metabolising hemicellulose and various pectin-associated glycans. Many of these plant glycans are believed to stimulate the metabolism and growth of specific bifidobacterial species and are for this reason classified as prebiotics. In this review, bifidobacterial carbohydrate metabolism, with a focus on plant poly-/oligosaccharide degradation and uptake, as well as its associated regulation, will be discussed.

## Introduction

Bifidobacteria are gut commensals and members of the Actinobacteria phylum harbouring genomes with a relatively high G + C content (considered approximately 50% or higher) (Ventura et al., 2007). They have been isolated from the gastrointestinal tract (GIT) of many mammalian species, including humans, as well as of insects and birds (Milani et al., 2016). Certain bifidobacterial strains or species, such as *Bifidobacterium longum* subsp. *longum*, are considered probiotic and are associated with various health benefits to the host, such as pathogen protection, including production of acetate to protect against enteropathogenic infection (Fukuda et al., 2011), sequestration of iron at the detriment of gut pathogens (Vazquez-Gutierrez et al., 2016), competing for epithelial binding sites with pathogens (Vazquez-Gutierrez et al., 2016), immune modulation through exopolysaccharide production (EPS) (Schiavi et al., 2016), alleviation of Irritable Bowel Syndrome (IBS) symptoms when supplied as a probiotic (Whorwell et al., 2006), and reducing the risk of contracting rotaviral diarrhea (Munoz et al., 2011). Bifidobacteria are known to metabolize a large number of glycans found in the gut environment. These glycans are metabolized through a unique pathway for carbohydrate fermentation which is termed the fructose-6-phosphoketolase (F6PK) pathway or ‘Bifid Shunt’ (de Vries and Stouthamer, 1967), which together with their distinctively high G + C content, above 50%, justified their taxonomic classification as a separate genus unrelated to lactic acid bacteria. The first bifidobacterial genome sequence, i.e., that of *B. longum* subsp. *longum* NCC2705, was published in 2002 (Schell et al., 2002), and its genome annotation reported a large number of genes dedicated to carbohydrate metabolism.

Bifidobacteria are highly prevalent in the infant gut and in particular the stool of breast-fed infants exhibit a significantly higher bifidobacterial abundance compared to their non-breast-fed counterparts (Bäckhed et al., 2015; Stewart et al., 2018). Human breast milk has been shown to contain viable bifidobacterial and is rich in so-called human milk oligosaccharides (HMOs) (Martín et al., 2009; Soto et al., 2014), which are highly specific growth substrates for particular bifidobacteria (Arboleya et al., 2011; James et al., 2016). It has also been found that the cessation of breast feeding and introduction to solid foods, referred to as weaning, is thought to induce changes to a more adult-like microbiome in infants (Bäckhed et al., 2015; Stewart et al., 2018). The relative abundance of bifidobacteria has been shown to decrease following weaning, and from adolescence into adulthood, with a further decline when their hosts become elderly (Hill et al., 2010; Odamaki et al., 2016).

Furthermore, the bifidobacterial species that are present in the human gut may vary depending on host age. One study reported that the *B*. *longum* subsp. *longum* taxon is associated with both the adult and infant gut, whilst *Bifidobacterium breve* is more frequently associated with the infant gut (Kato et al., 2017). In contrast, another study reported that *B*. *longum* subsp. *longum* and *B*. *breve* were associated with both the adult and infant gut (Turroni et al., 2009, 2012a; Odamaki et al., 2018). *Bifidobacterium dentium* has been found to be in higher abundance in the elderly gut although its natural niche is believed to be the oral cavity (Ouwehand et al., 2008). The type of sample taken for microbiota analysis, for instance colonic mucosal sample or stool sample, may determine which bifidobacterial species is more likely to be identified. However; another reason to explain why particular species of bifidobacteria are more prevalent and/or abundant in the infant or adult gut may be that they are specialized to metabolize specific dietary carbohydrates. For example, *B*. *breve* and *Bifidobacterium kashiwanohense* are generally capable of metabolising (certain) HMOs (Bunesova et al., 2016; James et al., 2016), whilst *B*. *longum* subsp. *longum* is specialized in the metabolism of particular plant glycans found in the adult diet (Schell et al., 2002; Riviere et al., 2018). *B*. *longum* subsp. *longum* strains have also been shown to encode members of glycosyl hydrolase (GH) families associated with the utilization of plant-derived carbohydrates (i.e., GH43, GH10, and GH5), reflecting their adaptation to plant glycan metabolism (Arboleya et al., 2018; Blanco et al., 2020).

A detailed understanding of carbohydrate metabolism of a particular bifidobacterial species and/or strains may offer opportunities to increase its abundance in the adult gut by dietary means. One way to positively modulate the gut microbiota is by the supplementation of so-called prebiotics, where a prebiotic is defined as ‘a substrate that is selectively utilized by host microorganisms conferring a health benefit’ (Gibson et al., 2017). Prebiotics that specifically stimulate bifidobacterial growth are termed ‘bifidogenic’ (Gibson and Roberfroid, 1995; Gibson et al., 1995). Knowledge on which plant carbohydrates can be metabolized by a bifidobacterial species/strain may therefore offer an opportunity to increase the abundance of bifidobacteria in the adult gut. For instance, *Bifidbacterium longum* subsp. *infantis* is associated with the infant gut, and is specialized in HMO metabolism, whilst *B*. *longum* subsp. *longum*, associated with both the infant and adult gut, can metabolize plant-derived oligosaccharides (O’Callaghan et al., 2015; Odamaki et al., 2018). This review will in particular focus on current knowledge regarding bifidobacterial plant-derived poly/oligosaccharide metabolism.

## The Plant Glycans Present in the Gut

**FIGURE 1:**
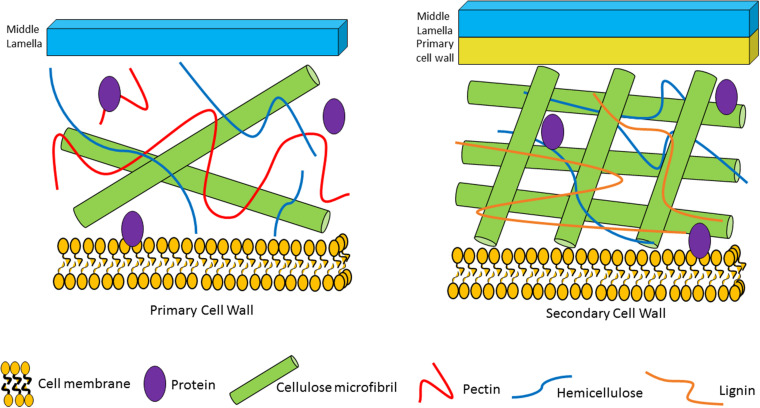
Plant cell wall composition and associated plant glycans/fibers. The primary cell wall is located outside of the plant plasma cell membrane. It is composed of cellulose microfibrils, hemicellulose and pectin. The secondary cell wall is located between the primary cell wall and the plasma cell wall. It consists of cellulose microfibrils, hemicellulose and lignin.

Dietary fibers/glycans are found in the plant cell wall (Figure 1; Koropatkin et al., 2012) and are common components in cereals (Broekaert et al., 2011; Shewry and Hey, 2015), fruit (van Laere et al., 2000; Posé et al., 2018), vegetables (Jonker et al., 2020; Klaassen and Trindade, 2020) and red grapes (Apolinar-Valiente et al., 2013), thus being a typical constituent of the human diet. Dietary fibers/glycans are metabolized by the gut microbiota in the large intestine (Flint et al., 2012). In contrast, meta-transcriptomic data from the microbiota in the small intestine shows, that phosphotransferase systems for simple sugars such as fructose, glucose and sucrose are utilized for carbohydrate metabolism suggesting that the small intestine microbiota utilize simpler sugars and not dietary fibers/glycans (Zoetendal et al., 2012). Some examples of dietary glycans are fructo-oligosaccharides (FOS), β-glucan, inulin, pectin, arabinoxylan, xylan, arabinan and starch (Holscher, 2017). Dietary fibers represent polymeric carbohydrates, including lignin, consisting of ten or more monomeric subunits that cannot be hydrolysed by enzymes found in the upper part of the human gastrointestinal tract (such as lactases, amylases and sucrases) (Alimentarius, 2010). Plant carbohydrate polymers with a size less than 10 monomeric subunits, but between a degree of polymerisation (DP) of 3 and 9, may in certain jurisdictions also be classified as dietary fibers (Alimentarius, 2010). Glycan is a much broader term that refers to a wide variety of carbohydrates (polymers and oligosaccharides). Glycans of dietary origin are generally indigestible to the human host, yet may be metabolized by the gut microbiota, and may include carbohydrates with less than 10 monomeric units that have been generated by the gut microbiota following dietary fiber degradation (Koropatkin et al., 2012).

**FIGURE 2:**
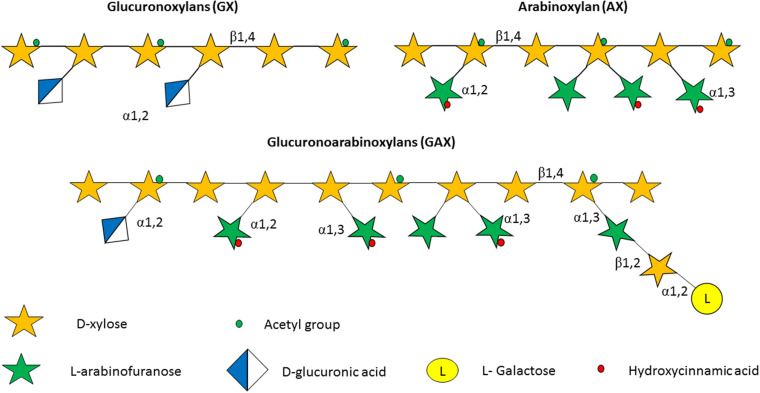
Structure of some of the hemicelluloses found in the plant cell wall. Hemicelluloses consist of a xylan backbone composed of β-1,4-linked D-xylose moieties, some of which may be substituted with an acetyl group. In glucoronoxylan (GX) the xylan backbone is substituted with D-glucuronic acid, while in the case of arabinoxylan (AX) the carbohydrate decorations consist of α-1,2-linked and/or α-1,3-linked arabinofuranose moieties. Finally, the glycan backbone of glucoronoarabinoxylan (GAX) possesses arabinose substitutions as in AX, in addition to D-glucuronic acid decorations that are α-1,2-linked to the xylan backbone, as well as D-xylose and L-galactose moieties that are β-1,2 linked and α-1,2-linked, respectively, to the arabinose substitutions.

**FIGURE 3:**
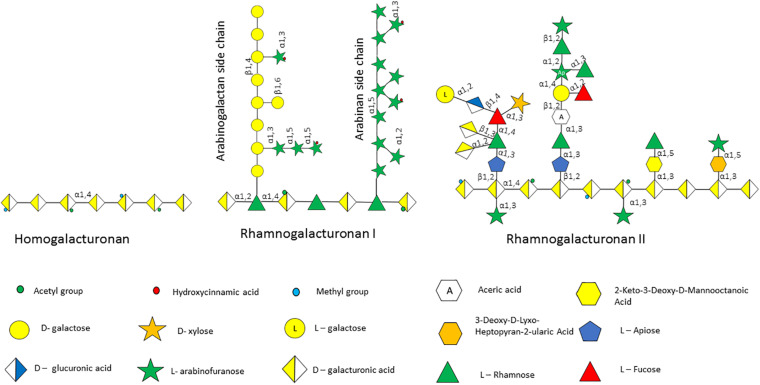
Pectin polysaccharides associated with the plant cell wall. Pectin is made up of several polysaccharides including homogalacturonan, rhamnogalacturonan I and rhamnogalacturonan II, the structure of which is schematically depicted.

The plant cell wall consists of a matrix comprized of cellulose fibrils, hemicellulose, pectin and lignin (Flint et al., 2012). Hemicelluloses are polysaccharides with β-1,4-linked backbones of xylose, mannose or glucose, to form (arabino)xylan (AX), mannan, and xyloglucan or β-glucan, respectively (Figure 2; Scheller and Ulvskov, 2010; Flint et al., 2012). In this review we focus on AX and arabinoxylo-oligosaccahrides (AXOS) metabolism. Lignin is predominantly composed of polymerised phenolic compounds such as hydroxycinnamic acids (HCA) (Struijs et al., 2008; Scheller and Ulvskov, 2010). Pectin is composed of various, highly variable polysaccharides including homogalacturonan (HG), xylogalacturonan, apiogalacturonan, rhamnogalacturonan I (RGI) and rhamnogalacturonan II (RGII) (Figure 3; Harholt et al., 2010). These pectic polysaccharides all contain an α-1,4-linked galacturonic acid backbone (Mohnen, 2008). HG is the simplest pectic polysaccharide, consisting of unsubstituted α-1,4-linked galacturonic acid moieties, whilst RGI is associated with an α-1,4-linked, D-galacturonic acid and rhamnose-containing backbone which can be substituted by other polymers such as galactan, arabinogalactan and arabinan (Anderson, 2015). RGII is the most complex chain, with a HG backbone that can be substituted with over twenty different glycosyl linkages and five different side chains (O’Neill et al., 2007). Both hemicellulose and pectic carbohydrates may also be decorated with HCAs such as ferulic acid or chlorogenic acid (Agger et al., 2010).

It is important to note that it is unlikely that in the gut bifidobacteria can metabolize these large, mostly insoluble complex plant polysaccharides on their own; however, they may be able to utilize specific components and/or side chains of these glycans. Alternatively, it is possible that these plant-derived polysaccharides arrive in the large intestine undigested, where they are degraded by particular, so-called keystone species. Examples of such keystone species include *Bacteroides cellulosilyticus, Bacteroides caccae*, and *Dysgonomonas gadei*, which for example are capable of the degradation of type II arabinogalactan due to their extracellular endo-β-1,3-galactanase activity (Cartmell et al., 2018). This extracellular degradation allows the release of soluble oligosaccharides, such as arabino-oligosaccharides (AOS), AXOS, and galacto-oligosaccharides which may then become available as metabolic ‘cross-feeding’ substrates for other gut commensals, such as bifidobacteria. For example, *B. breve* UCC2003 can cross-feed on certain galacto-oligosaccharides released from larch wood arabinogalactan by *Ba*. *cellulosilyticus* (Munoz et al., 2020). Therefore, the current definition of a prebiotic does not include glycans, such as intact pectin or xylan, which may stimulate growth of a broad range of species in the GIT (Gibson et al., 2017). Knowledge on carbohydrate metabolism of bifidobacteria can therefore be exploited to develop prebiotics and/or ‘synbiotics’ [a combination of a probiotic organism and a corresponding prebiotic that selectively stimulates growth of the administered probiotic, and therefore its associated beneficial effect(s)] (van Zanten et al., 2012; Kearney and Gibbons, 2018; Swanson et al., 2020).

## The Bifid Shunt – a Unique Carbohydrate Metabolic Pathway

As mentioned above, bifidobacteria possess a unique pathway for carbohydrate assimilation which is termed the F6PK pathway (de Vries and Stouthamer, 1967, 1968). This complex pathway, with its key enzyme fructose-6-phosphoketolase, is very distinct from the homofermentative (Embden-Meyerhof-Parnas) or heterofermentative (phosphoketolase or pentose phosphate) glycolytic pathways (Macfarlane and Macfarlane, 2003; Mayo and van Sinderen, 2010) and is exclusively found in the *Bifidobacteriaceae* family and members of the *Coriobacteriales* order (Palframan et al., 2003; Killer et al., 2010; Gupta et al., 2017). The F6PK pathway can assimilate both hexose and pentose sugars by fermentation into lactate and acetate (Egan and Van Sinderen, 2018), with a theoretical yield of 1.5 mol acetate and 1 mol of lactate for every mol of glucose consumed (de Vries and Stouthamer, 1967; Wolin et al., 1998), or a 1:1 ratio of lactate and acetate in the case of pentose sugar fermentation (Palframan et al., 2003). Hexose sugars are fed into the F6PK pathway as fructose-6-phosphate whilst pentose sugars can enter the pathway as ribulose-5-phosphate or xylulose-5-phosphate (Egan and Van Sinderen, 2018). However, the actual ratio of acetate to lactate produced depends on various factors including the individual strain, pH and growth rate, which in turn differs depending on the carbohydrate substrate utilized (Palframan et al., 2003; Watson et al., 2013; McLaughlin et al., 2015). The short chain fatty acid (SCFA) acetate, when produced by *B. longum* subsp. *longum* from fructose fermentation, has been shown to generate anti-inflammatory effects and/or to block epithelial apoptosis in a murine model, thereby preventing translocation of the Shiga toxin produced by *Escherichia coli* O157:H7 into the bloodstream, and in this way providing protection against this gut pathogen (Fukuda et al., 2011). In addition, lactate, an organic acid (but not a SCFA), has also been shown to have a direct effect on enterocyte proliferation and contributes to hyperproliferation of enterocytes after starvation in a mouse model thus supporting intestinal barrier integrity (Okada et al., 2013). The F6PK pathway theoretically produces 2.5 molecules of ATP per 1 metabolized glucose molecule, which is higher than the energy yield of homofermentation by lactobacilli species at 2 molecules of ATP per 1 molecule of glucose metabolized (Palframan et al., 2003).

## Bifidobacterial Carbohydrate Import

Bifidobacteria are capable of metabolizing a diverse range of mono-, di-, and oligo-saccharides found in the GIT environment, which they mainly import into their cytoplasm by means of so-called ABC-type (ATP-binding cassette) transporters or major facilitator superfamily (MFS) transport systems, such as proton symporters and proton-motive force-driven permeases (Schell et al., 2002; Pokusaeva et al., 2011a). Furthermore, most bifidobacterial species encode Phosphoenol Pyruvate-Phosphotransferase Systems (PEP-PTSs) (Maze et al., 2007; Turroni et al., 2012b). However, ABC-type transporters generally are the most commonly employed systems to internalize carbohydrates in bifidobacteria. For example, *B*. *longum* subsp. *longum* NCC2705 is predicted to encode 13 ABC type transporters, 3 MFS transporters, 1 PTS system, 1 glycoside pentoside cation symporter family transporter (GPH) and 1 major intrinsic protein family (MIP) transporter (Parche et al., 2007). Similarly, *Bifidobacterium longum* subsp. *infantis* ATCC15697 is predicted to encode 13 ABC transporter systems (Sela et al., 2008). However, there are exceptions; as a representative of its species *Bifidobacterium bifidum* PRL2010 preferentially utilizes PEP-PTS systems to import carbohydrates, most likely because this strain degrades complex carbohydrates extracellularly, thereby releasing mostly monosaccharides, explaining why PRL2010 encodes just two ABC-type transporters and four PEP-PTS systems (Turroni et al., 2012b). ABC-type transporters hydrolyse ATP in order to import their substrate, such as a carbohydrate, against a chemical gradient (Wilkens, 2015). An ABC-type transport system typically consists of two transmembrane-associated proteins, which act as permeases to translocate the substrate across the membrane and two ATP-binding proteins that provide the energy required for transport (Rees et al., 2009). The so-called substrate binding protein (SBP) binds a specific carbohydrate monomer or oligosaccharide (or very related substrates) and brings the substrate to the permease to be imported (Rees et al., 2009). This can affect the growth rate of a strain; for instance, the SBP of an ABC-type transporter specified by *Bifidobacterium animalis* subsp. *lactis* B1-04 binds preferentially to β-1,6-galactobiose over β-1,4-galactobiose, and this may in part contribute to faster growth of this strain on the former substrate (Theilmann et al., 2019). The heavy reliance on carbohydrate-specific ABC-type transporters by bifidobacteria for internalization of their carbon and energy sources may reflect the need for members of this genus to be versatile in metabolizing a diverse range of carbohydrates, including various plant-derived oligosaccharides present in the gut environment (Schneider, 2001; Chandravanshi et al., 2019), rather than relying on PEP-PTSs, which are mainly restricted to monosaccharide utilization (Deutscher et al., 2006). For example, an ABC-type transporter was found to confer the ability of *B*. *animalis* subsp. *lactis* B1-04 to metabolize the tri-saccharide raffinose (and related oligosaccharides) and this strain was able to outcompete *Bacteroides ovatus* when both strains are co-cultured on raffinose (Ejby et al., 2016).

## Enzymatic Degradation of Plant-Oligosaccharides by Bifidobacteria

A relatively high percentage, 13.7%, of the overall *Bifidobacterium* pan-genome is dedicated to carbohydrate metabolism (Milani et al., 2014, 2016), and a similar percentage, 13.23 and 12.5%, when representative genomes of *B*. *breve* and *B*. *longum* subsp. *longum*, respectively, are scrutinized (Bottacini et al., 2014; O’Callaghan et al., 2015). However, just 5.5% of the *Bifidobacterium* core genome (i.e., genus-wide conserved genes) is dedicated to carbohydrate metabolic pathways suggesting that in order to survive in the GIT environment the acquisition of carbohydrate metabolic genes in the accessory genome is important (Milani et al., 2014). This is not surprising considering the wide diversity of carbohydrates that bifidobacteria may encounter in the GIT environment. Bifidobacteria, like other members of the gut microbiota, possess ‘Carbohydrate Active Enzymes’ (CAZymes), such as GHs that break the glycosidic bonds between carbohydrate moieties and covalent linkages between carbohydrates and non-carbohydrate moieties. Carbohydrate esterases (CE) cleave the ester bound between a HCA and a carbohydrate residue, and thereby may provide access to other GHs to hydrolyse plant-derived oligosaccharides (Kelly et al., 2018).

**FIGURE 4:**
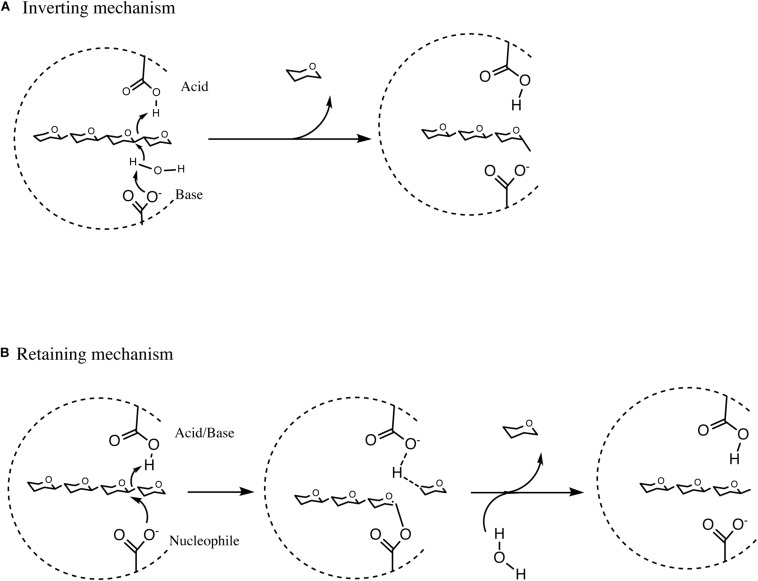
Summary of Inverting hydrolysis, retaining hydrolysis and transglycosylation. **(A)** Summary of inverting single displacement mechanism. **(B)** Summary of retaining double displacement mechanism. See text for details of the reactions.

The process of hydrolysis by GHs can occur by two distinct routes, either (i) by means of a single displacement mechanism which takes place in a single step and which results in the inversion of the anomeric centre, or (ii) by a double displacement mechanism involving two catalytic steps resulting in the retention of the anomeric center following hydrolysis (Davies and Henrissat, 1995; Withers, 2001). Hydrolysis of a glycosidic linkage between two monosaccharides is usually mediated by two catalytic carboxylic residues in the corresponding GH, one being a proton donor represented by an acid, while the other acting as a proton acceptor and represented by a base, activating a water molecule that acts as a nucleophile, in the inverting enzyme (van den Broek et al., 2008). However, in the retaining configuration, one carboxylic residue acts as an acid/base and another as nucleophile (Figure 4; Davies and Henrissat, 1995).

In the first step of the double displacement mechanism one residue protonates the glycosidic oxygen leading to the hydrolysis of the glycolytic bond and the formation of an oxocarbenium ion-like transition state. A glycosyl-enzyme intermediate is then formed by the other residue (nucleophile) attacking the anomeric center of the sugar. In the second step of the reaction, termed deglycosylation, the basic residue deprotonates a water molecule which in turn attacks the glycosyl-enzyme intermediate to cause hydrolysis of the glycosyl-enzyme intermediate (Figure 4; Withers, 2001; van den Broek et al., 2008). CAZymes can either degrade oligo- or polysaccharides at the end of the molecule, most commonly from the non-reducing end, or in between individual saccharidic moieties, representing hydrolytic abilities that are referred to as exo or endo activity, respectively (Mangas-Sánchez and Adlercreutz, 2015). The remainder of this review will focus on bifidobacterial GHs and CEs involved in the degradation of a selected number of plant-derived poly- and oligo-saccharides.

## Xylan and Xylo-oligosaccahrides (XOS)

**FIGURE 5:**
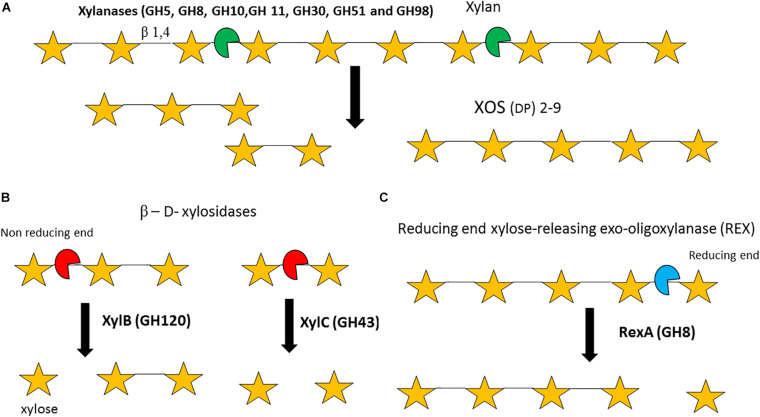
Enzymatic degradation of xylan and xylo-oligosacharides (XOS). Degradation of the xylan backbone to XOS by endo-xylanases **(A)**. Degradation of XOS by β-D-xylosidases **(B)**. Degradation of XOS by a ‘Reducing end xylose releasing exo-oligoxylanase **(C)**. DP = degree of polymerization. Enzyme names are indicated in bold. See text for details.

Bifidobacteria are capable of growth on several plant-derived poly/oligo-saccharides and their derived monomers (Watson et al., 2013; McLaughlin et al., 2015). Specifically, the *B*. *longum* subsp. *longum* and *Bifidobacterium adolescentis* taxa seem to be particularly well adapted to plant-based carbohydrate utilisation (O’Callaghan et al., 2015). Hemicelluloses include carbohydrates that generally possess a β-1,4-linked backbone, for example xylan, which is composed of β-1,4-linked D-xylose moieties (Scheller and Ulvskov, 2010). Furthermore, this xylan backbone can be decorated or substituted with L- or D-arabinose, xylose, galactose and D-glucuronic acid (Ndeh and Gilbert, 2018). Based on the nature of these substituents xylan is further categorized into AX, glucuronoxylans (GX) and glucuronoarabinoxylans (GAX) (Rogowski et al., 2015). AX from corn may also contain α-1,2-linked galactose to arabinose side chains (Appeldoorn et al., 2010; Pollet et al., 2012; Figure 5).

It should be noted that the xylan backbone typically requires removal of its substitutions before it can be degraded, a process that may involve multiple enzymatic activities. Xylanases or endo-1,4-β-xylanases (EC 3.2.1.8, GH5, GH8, GH10, GH11, GH30, GH51, and GH98) are endo-acting enzymes that randomly hydrolyse the internal β-1,4 bond between D-xylose residues within a xylan polymer to produce XOS (with a degree of polymerisation ranging between two and nine) (Figure 5A; Collins et al., 2005). Currently, no bifidobacterial strain/species is known to be able to grow on the polymeric, insoluble xylan backbone. Therefore, it is likely that in the GIT species such as *Ba*. *ovatus*, *Bacteroides xylanisolvens* or *Bacteroides intestinalis* degrade the xylan backbone into soluble XOS, which then becomes available for other species to utilize (Zhang et al., 2014; Despres et al., 2016; Wang et al., 2016). Particular bifidobacterial species, e.g., *B*. *longum* subsp. *longum* and *B. adolescentis*, are able to metabolize xylan-derived XOS (Falck et al., 2013; Arboleya et al., 2018) and several enzymes have been implicated in the degradation of this oligomeric substrate by bifidobacteria. β- D-xylosidases (EC 3.2.1.7, GH1, GH2, GH3, GH43, GH51, GH52, GH54, GH116, and GH120) are exo-enzymes which can hydrolyse XOS starting at the non-reducing xylose residue. For instance, a β-1,4 xylosidase (EC 3.2.1.37) (GH51) from *B*. *breve* K-110 was shown to elicit activity against *p*-Nitrophenyl (*p*Np) β-D-xylopyranoside, yet exhibits very limited activity against xylan (Shin et al., 2003). Furthermore, *B*. *adolescentis* LMG10502 encodes two β-xylosidases: XylB (GH120) which hydrolyses XOS but not xylobiose, and XylC (GH43), which hydrolyses xylobiose (Lagaert et al., 2011; Figure 5B). In addition, the GH8 RexA or reducing-end, xylose-releasing exo-oligoxylanase enzyme (EC 3.2.1.156) (Valenzuela et al., 2016) from *B. adolescentis* LMG10502 was shown to elicit limited activity against xylan, no activity against xylobiose or *p*Np-β-D-xylopyranoside, though was shown to exhibit activity against XOS with a DP of 3 and above (Figure 5C; Lagaert et al., 2007).

Transcriptional and proteome analysis of *B*. *animalis* subsp. *lactis* BB-12 grown on XOS revealed expression of a number of xylanases, β-xylosidases and ABC transporters (Lagaert et al., 2007). Bifidobacterial species/strains that are able to utilize XOS, such as *B*. *longum* subsp. *longum* and *B. adolescentis*, usually metabolize XOS only up to a DP of six, i.e. xylohexose due to size limitations of the corresponding XOS transport system (Wang et al., 2010; Amaretti et al., 2013). It must be noted that generally bifidobacterial CAZymes act intracellularly, although extracellular hydrolysis of XOS by an apparently extracellular bifidobacterial β-1,4 xylosidase has been reported for *B*. *adolescentis* (Amaretti et al., 2013).

## AX, AXOS, Arabinan, Arabinogalactan, and Corn GAX

The xylose residues in xylan and XOS can be mono-substituted with L-arabinose at the C(O)2 or C(O)3 positions or di-substituted with L-arabinose at both C(O)2 and C(O)3 positions, while these arabinose substitutions can either be α-1,2-linked or α-1,3-linked (Figure 2; Scheller and Ulvskov, 2010; De Vuyst et al., 2014). Only a limited number of bifidobacterial species/strains, e.g., *B*. *longum* subsp. *longum*, are able to metabolize such AX and AXOS glycans (O’Callaghan et al., 2015; Riviere et al., 2015; Truchado et al., 2015). Depending on the particular bifidobacterial species/strain different components of AX or AXOS are utilized. One study classified bifidobacterial species/strains into five clusters based on the particular AX, AXOS, or XOS components a given strain could metabolize: cluster I, metabolism of monosaccharides arabinose and xylose, but no metabolism of XOS or arabinose substituents; cluster II, metabolism of mono- or di-substituted arabinose, yet no utilization of the XOS backbone; cluster III, utilization of the XOS backbone but no utilization of arabinose substituents; cluster IV, utilization of both arabinose substituents and XOS, up to xylotetraose of AXOS; cluster V, utilization of AXOS including up to xylohexose XOS chains (Riviere et al., 2014). Therefore, the presence of AX, AXOS, and XOS in the GIT supports growth of various bifidobacterial species/strains either directly or indirectly through possible cross-feeding activities (De Vuyst et al., 2014). In this sense, *Ba. ovatus* has been shown to support growth of *B. adolescentis* when they interact on simple xylans, such as wheat AX and birch GX (Rogowski et al., 2015). However, *Ba. ovatus* cannot cross-feed with *Bifidobacterium* sp. when they use complex dietary xylans, such as corn AX. The reason for this inability is that *Bifidobacterium* sp. lack the catalytic apparatus needed to metabolize the oligosaccharides released from complex dietary xylans by *Ba*. *ovatus*. This is consistent with the fact that *B. adolescentis* is unable to metabolize corn AX, even if it is pretreated with the GHs located on the surface of *Ba. ovatus* (Rogowski et al., 2015).

Pectin is composed of multiple complex glycans that can be utilized by the gut microbiota (Ndeh et al., 2017; Luis et al., 2018). Probably because of its complexity there are currently no known bifidobacterial species that are able to directly metabolize pectin (Figure 3). It is therefore presumed that other gut commensals such as *Bacteroides thetaiotaomicron* degrade these large polymers extracellularly and that certain bifidobacterial species can then scavenge the released mono- and oligosaccharides, as shown previously by co-cultivation of *B*. *longum* subsp. *longum* with *Ba*. *thetaiotaomicron* in the presence of arabinogalactan (Degnan and Macfarlane, 1995). *B*. *breve* UCC2003 can cross-feed on β-1,3 galacto-di/trisaccharides released from larchwood arabinogalactan by *Ba. cellulosilyticus* (Munoz et al., 2020). *B. longum* subsp. *longum* strains have been shown to grow on the pectic components arabinan and arabinogalactan (O’Connell Motherway et al., 2011; Komeno et al., 2019). Arabinan consists of an α-1,5-linked L-arabinose backbone that can be mono- or di-substituted with either α-1,2-linked and/or α-1,3-linked L-arabinose (Mohnen, 2008). Type I arabinogalactan is usually linked to other pectin-associated glycans, whereas type II arabinogalactan is O-linked to a protein backbone. Both arabinogalactan types are key components of the plant cell wall (Seifert and Roberts, 2007; Sakamoto and Ishimaru, 2013). Type I arabinogalactan is composed of a β-1,4-linked D-galactose backbone substituted by α-1,5-linked L-arabinose, while type II arabinogalactan is composed of a β-1,3-linked D-galactose backbone that can be substituted with α-1,3-linked arabinose and α-1,6-linked galactose side chains with further decorations with other minor monosaccharide components, such as rhamnose, (methyl)glucuronic acid, xylose or fucose (Mohnen, 2008; Sakamoto and Ishimaru, 2013; Cartmell et al., 2018).

α-L-arabinofuranosidases (EC 3.2.1.55, GH1, GH2, GH3, GH5, GH39, GH43, GH51, GH54, and GH62) are exo-acting enzymes that can cleave arabinose moieties from the polymeric backbone of xylan, XOS, galactan or arabinan/AOS (Margolles and de los Reyes-Gavilan, 2003; Lagaert et al., 2014). Arabinofuranosidases typically remove mono-substituted α-1,2 linked and/or α-1,3 linked arabinose from their particular substrate backbone (van den Broek et al., 2005; Bourgois et al., 2007), although certain arabinofuranosidases are specialized in removing arabinose from a di-substituted substrate (van den Broek et al., 2005; Cartmell et al., 2011). The ability to degrade AXOS has been shown to be species/strain dependent and certain bifidobacterial species/strains are only able to metabolize the arabinose substitutions on XOS (Riviere et al., 2014). An α-arabinofuranosidase (GH51) produced by *B*. *longum* subsp. *longum* has been shown to release arabinose from AX (Margolles and de los Reyes-Gavilan, 2003), while AbfA (GH43) from *B*. *adolescentis* was shown to remove arabinose residues from the C(O)2 and C(O)3 positions of mono-substituted xylose, and AbfB (GH51) and AXHd3 (GH43) were demonstrated to release arabinose residues from the C(O)3 of disubstituted xylose residues (van den Broek et al., 2005; Lagaert et al., 2010). L-arabinofuranosidases can also act as exo-enzymes on AOS present in arabinan or arabinogalactan. For example, an α-L-arabinofuranosidase (GH1) from *B*. *adolescentis* was shown to possess exo-activity on α-1,5-linked AOS (DP 2-5) (Suzuki et al., 2013). Similarly, the *B*. *longum* subsp. *longum* ArafC (GH43) was shown to be capable of removing α-1,2-linked and α-1,3-linked arabinose side chains of AX and arabinan, yet ArafD (GH43) was shown to exhibit hydrolytic activity towards α-1,5-linked arabinan (Komeno et al., 2019). α-L-arabinofuranosidases can also release arabinose side chains from galactose residues in arabinogalacatan; BlArafA (GH43), an α-arabinofuranosidase produced by *B*. *longum* subsp. *longum*, can release α-1,3-linked arabinose from β-1,6-galacto-oligosaccharides (Fujita et al., 2019). Endo-α-arabinases (EC 3.2.1.99) hydrolyse the α-1,5-linkage within the arabinan backbone (Arnal et al., 2015) and it is likely that arabinofuranosidases must first remove the L-arabinose substituents before the backbone can be effectively cleaved. Currently, no endo-arabinases have been described in bifidobacteria. β-L-arabinofuranosidases (EC 3.2.1.185, GH127, GH142, and GH146) remove β-linked arabinose substitutions from plant-oligosaccharides; β-linkages are less common and found on extensins (proteoglycans that are abundant in carrots) type II arabinogalactan, RGI and RGII (from pectin polysaccharides) linked to plant cell wall proteins (Lansky et al., 2014; Ndeh et al., 2017; Luis et al., 2018). In *B*. *longum* subsp. *longum*, β-arabinofuranosidases HypBA1 (GH127) and HypBA2 (GH121) release arabinose from β-1,2-linked arabinosaccharides (DP 2-3) linked to hyproxyline (Fujita et al., 2011, 2014b). Several bifidobacterial α-L-arabinofuranosidases and β-L-arabinofuranosidases have been reported in literature and their salient features are summarized in Table 1.

**TABLE 1:** Summary of characterised bifidobacterial arabinofuranosidases.

Enzyme name/classification	Substrates	GH family	References	Species
AbfB	Arabinan, AX, arabinobiose - arabinopentose	GH51	Margolles and de los Reyes-Gavilan, 2003	*B*. *ll*
α-L-arabinofuranosidase				
BXA43	XOS (DP 2-4)	GH43	Viborg et al., 2013	*B*. *al*
β xylosidase/ α-L-arabinofuranosidase	*p*NP-α-L araf			
	*p*Np-β-D Xyl			
BAD0156	pNP-α-L araf	GH1	Suzuki et al., 2013	*B*. *a*
α-L-arabinofuranosidase	α-1,5 arabinosaccharides			
BlArafC	*p*NP-α-L araf	GH43	Komeno et al., 2019	*B*. *ll*
α-L-arabinofuranosidase	Arabinan			
	AX			
BlArafD	*p*NP-α-L araf	GH43	Komeno et al., 2019	*B*. *ll*
α-L-arabinofuranosidase	Arabinan			
BlArafA	*p*NP α L araf	GH43	Fujita et al., 2019	*B*. *ll*
α-L-arabinofuranosidase	α-1,3-Ara*f* Gal_3_			
	Ara*f*-α-1,3-Ara*f*-α-OMe			
	Radish AG			
	Larch AG			
	Arabinan			
Blon_0625	*p*NP-α-L araf	GH3	Matsumoto et al., 2015	*B*. *li*
α-L-arabinofuranosidase				
HypBA2	β1,2-Arabinose hyproxyline	GH121	Fujita et al., 2011	*B*. *ll*
β-L-arabinofuranosidase				
	β1,2-linked arabinotriose - hyproxyline			
	Arabinan			
	Debranched Arabinan			
HyBA1	β 1,2-linked Arabinose – hyproxyline (DP 2 and 3)	GH127	Fujita et al., 2014b; Ito et al., 2014; Zhu et al., 2014	*B*. *ll*
β-L-arabinofuranosidase				
	Arabinobiose - ME			
AfuB-H1	*p*NP-α-L araf	GH51	Lee et al., 2011	*B*. *ll*
α-L-arabinofuranosidase				
AbfA	AX	GH43	Lagaert et al., 2010	*B*. *a*
α-L-arabinofuranosidase	AXOS			
	*p*NP-α-L araf			
	*p*NP-β-Xyl			
AbfB	AX,	GH43	Lagaert et al., 2010	*B*. *a*
α-L-arabinofuranosidase	AXOS			
	*p*NP-α-L araf			
	Arabinan			
AXH-d3	AX	GH43	van den Broek et al., 2005	*B*. *a*
	AXOS			
	Arabinan			
α-L- arabinofuranosidase	*p*NP-α-L araf	–	Shin et al., 2003	*B. b*
	Ginsenoside RC			

*L araf, L-arabinofuranose; D Xyl, D-xylopyranoside; ME, methyl group; Gal, galactose; OMe, O-linked methyl group; AG, arabinogalactan; AX, Arabinoxylan; AXOS, arabinoxylo-oligosaccharides; B. ll, Bifidobacterium longum subsp. longum; B. al, Bifidobacterium animalis subsp. lactis; B.b, Bifidobacterium breve; B. a, Bifidobacterium adolescentis; B. li, Bifidobacterium longum subsp. infantis.*

**FIGURE 6:**
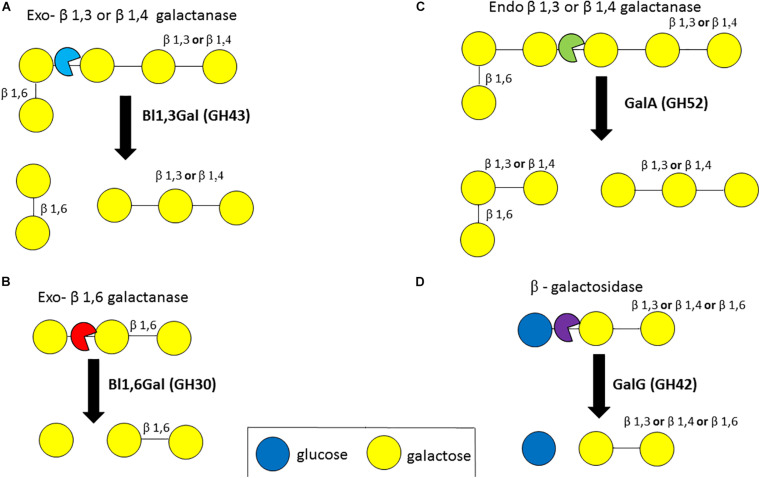
Enzymatic degradation of galactan. Degradation of galactan by exo-β1,3- or β1,4-galactanases **(A)**. Degradation of galactan by exo-β1,6-galactanases **(B)**. Degradation of galactan by endo-β1,3- or β1,4-galactanases **(C)**. Degradation of a galactose-sugar moiety bond by β-galactosidases **(D)**. Enzyme names are indicated in bold. See text for details.

Various enzymes are required to degrade plant-derived galactan. Exo-acting β-1,3-galactanases (EC 3.2.1.145, GH43 subfamily 24) cleave the β-1,3-D-galactose backbone of type II arabinogalactan even in the presence of β-1,6-D galactose side chains through a by-pass mechanism (Ichinose et al., 2006; Cartmell et al., 2018). Exo-acting β-1,4-galactanases (no designated EC number) cleave terminal β-1,4-linked galactose bonds (Sakamoto and Ishimaru, 2013). An exo-β-1,3 galactanase (GH43 subfamily 24), (Bl1,3Gal) isolated from *B*. *longum* subsp. *longum* was shown to hydrolyse β-1,3-linked galacto-oligosaccharides (DP between 2 and 5) and de-arabinosylated larchwood arabinogalactan (Fujita et al., 2014a). This Bl1,3Gal enzyme is unusual as it exhibits a higher activity for β-1,3-galactan when the latter substrate is substituted with β-1,6-side chains, apparently recognizing these side chains as a specificity determinant in the active site. Similarly, BgaA (GH2) of *B*. *breve* UCC2003 was shown to cleave β-1,3-linked galactobiose/triose (Figure 6A) (Munoz et al., 2020). An exo-β-1,6-galactobiohydrolase (Bl1,6-Gal, GH30) from the same species was shown to degrade β-1,6-linked galactose (DP between 2 and 4) and β-1,6-galactan, but was not able to degrade arabinose substituted substrates (Fujita et al., 2019; Figure 6B). Furthermore, depending on the linkage type of the galactan backbone degradation may involve endo-acting β-1,3-galactanases (EC 3.2.1.181, GH30) (Sakamoto and Ishimaru, 2013), β-1,4 galactanases (EC 3.2.1.89, GH53) (Zavaleta and Eyzaguirre, 2016) or β-1,6-galactanases (EC 3.2.1.164, GH30) (Sakamoto and Ishimaru, 2013).

In *B. longum* subsp. *longum*, an extracellular endo-acting β-galactanase, designated GalA, was found to be capable of cleaving β-1,4 and β-1,3-galactan linkages (Hinz et al., 2005; Figure 6C). The extracellular GalA (GH52) homolog in *B*. *breve* UCC2003, which is present in certain strains of this species, was found to elicit hydrolytic activity towards galactan, thereby releasing galacto-oligosaccharides (O’Connell Motherway et al., 2011). GalA is encoded by a galactan utilization cluster in both *B*. *breve* UCC2003 and *B*. *longum* subsp. *longum* strains, and in addition specifies an ABC type transporter, and GalG (GH42), a β-galactosidase (O’Connell Motherway et al., 2011, 2013). β-galactosidases (EC 3.2.1.23, GH1, GH2, GH35, GH39, GH42, GH59, GH147, and GH165) hydrolyse linkages between a galactose moiety and another sugar moiety and several β-galactosidases have been identified in *B*. *bifidum*, *B. longum* subsp. *longum*, *B*. *longum* subsp. *infantis* and *B*. *breve*, being able to hydrolyse β-1,3, β-1,4 or β-1,6 linkages in galacto-oligosaccahrides and HMO substrates (Goulas et al., 2007; Godoy et al., 2016; James et al., 2016; Sotoya et al., 2017; Ambrogi et al., 2019; Figure 6D).

Finally, the backbone or side chains of these plant-derived oligomers may also be substituted with HCAs. HCAs that are in free form are absorbed by the small intestine (Cremin et al., 2001), whereas HCAs that are linked to plant-derived polysaccharides are not readily absorbed in the small intestine and are therefore likely to reach the colon (Clifford, 2004). Many hemicelluloses and pectic plant polymers have HCAs attached by an ester bond to the (O)5 position of the sugar moiety (Saulnier and Thibault, 1999; Scheller and Ulvskov, 2010). HCA-specific esterases (EC 3.1.1.73, CE1 and CE6) catalyse the hydrolysis of the ester bond between a given HCA, for example ferulic acid and *p-*coumaric acid, and a sugar moiety (arabinose, galactose or xylose) on AX and pectin plant-oligomers (Wong, 2006). These HCA-specific esterases possess an alpha/beta hydrolase fold, a consensus motif (Gly-X-Ser-X-Gly) and a catalytic triad consisting of Ser-His-Asp residues (Bornscheuer, 2002). Bifidobacterial esterases active against HCAs have been described, including the CaeA esterase in *B*. *longum* subsp. *longum*, whose encoding gene is located within the same genetic locus as the genes encoding GH enzymes that are predicted to be involved in plant-oligosaccharide utilization (Raimondi et al., 2015; Fritsch et al., 2017; Kelly et al., 2018).

## Regulation of Carbohydrate Metabolism in Bifidobacteria

Carbon catabolite repression (CCR) refers to a global regulatory mechanism by which bacteria can preferentially metabolize the ‘optimal’ carbon source that has the greatest energy yield, amongst a mixture of carbon sources, and involves inhibition of the metabolic pathways of the less preferred carbon sources (Stülke and Hillen, 1999). This is important in the GIT environment where potentially multiple carbohydrate sources are present and the optimal carbon source must be consumed to increase chances of survival in the gut. There are many mechanisms of CCR and this can vary from species to species. For instance, CCR may involve transcriptional activation, transcriptional repression and/or translational regulation (Görke and Stülke, 2008). In the CCR paradigm, many bacteria, such as *Escherichia coli*, the ‘optimal’ substrate glucose is metabolized preferentially (Inada et al., 1996). Certain bacteria, e.g., *B*. *longum* subsp. *longum* and *Streptococcus thermophilus*, preferentially metabolize lactose over glucose (van den Bogaard et al., 2000; Kim et al., 2003; Parche et al., 2006). The preference of other sugars over glucose for metabolism is also termed reverse CCR (Görke and Stülke, 2008). CCR-resembling regulation has previously been described in bifidobacteria. In particular, in *B*. *breve* UCC2003, a FOS utilization cluster inducible by growth on sucrose or Actilight, a commercial FOS prebiotic, was shown to be downregulated in the presence of glucose and/or fructose – sucrose mixes (Ryan et al., 2005). CCR may be important from an ecological perspective, as it may avoid species/strain competition for limited carbon sources in the gut environment (Brückner and Titgemeyer, 2002). However, CCR is not the only model to describe the regulation of carbohydrate metabolism in bacteria. Indeed, *B*. *breve* and *Corynebacterium glutamicum*, both members of the Actinobacteria phylum, have been shown to globally regulate their central metabolic flux and control co-metabolism of multiple sugars (Wendisch et al., 2000; Lanigan et al., 2019).

LacI-type transcriptional regulators are the most prevalent and abundant family of bifidobacterial TFs; in one study they were shown to account for 63% of all identified regulators encoded by ten bifidobacterial genomes (Khoroshkin et al., 2016). LacI-type transcriptional factors in bifidobacteria typically act as carbohydrate-specific transcriptional repressors and are therefore important allowing only appropriate expression of carbohydrate metabolism genes in the presence of the corresponding saccharidic substrate in the GIT environment. LacI-type transcription factors contain a so-called helix-turn-helix DNA binding domain at their N-terminus, a core domain to bind sugar ligands and a multimerization domain for the formation of dimers and/or tetramers (Lewis et al., 1996).

Bifidobacterial LacI-type transcriptional factors have been shown, *in silico* and *in vitro*, at a local level to control genes and/or operons involved in carbohydrate metabolism for various carbohydrates including HMOs (James et al., 2018), galactan (O’Connell Motherway et al., 2011), melezitose (O’Connell et al., 2014), AOS (Arzamasov et al., 2018), FOS (Ryan et al., 2005), ribose (Pokusaeva et al., 2010) and cellodextrin (Pokusaeva et al., 2011b). Nonetheless, other types of transcriptional regulation have been reported to be involved in transcriptional control of genes involved in carbohydrate metabolism. Examples are represented by a GntR-type transcription factor (TF) for sialic acid utilization (Egan et al., 2015), a so-called repressor open reading frame kinase (or ROK) TF for raffinose and stachyose metabolism (O’Connell et al., 2014), and a NagC/XylR-type repressor involved in sulfated sugar metabolism regulation (Egan et al., 2016). Therefore, LacI-type and other transcriptional regulator families play an important role in the regulation of bifidobacterial carbohydrate metabolism.

Central carbohydrate metabolism in bifidobacteria is represented by the ‘Bifid Shunt’, which is regulated by two LacI-type regulators, designated AraQ and MalR1 (Lanigan et al., 2019), employing a mechanism that is reminiscent to that reported for *C*. *glutamicum* (Wendisch et al., 2000). This mechanism of global carbohydrate regulation may be advantageous to bifidobacteria in the GIT environment by allowing these gut commensals to quickly and effectively respond to the various glycans that can be present in the GIT at any given time.

## *B*. *longum* subsp. *longum*, an Infant and Adult Associated Bifidobacterial Species

*B*. *longum* subsp. *longum* is a bifidobacterial species that is associated with both the infant and adult gut microbiota (Odamaki et al., 2018), while *B*. *longum* subsp. *infantis* is typically associated with the infant gut (Turroni et al., 2012a). As mentioned above a major factor that influences the bifidobacterial species composition in the infant or adult gut is the nature of the carbohydrates present in the diet, though other factors may also affect the ability of bifidobacteria to colonize and survive in the gut environment, as reviewed elsewhere (González-Rodríguez et al., 2013). In the infant gut and unaffected by host enzymes, HMOs are the main dietary glycans and these are mainly composed of hexose sugars; for example, most HMOs contain N-acetylglucosamine (GlcNac) and β1,3- or β1,4-linked lacto-N-biose (LNB, Galβ1,3GlcNac) residues with a terminal lactose at the reducing end (Smilowitz et al., 2014), and are frequently decorated with fucose or sialic acid (Bode and Jantscher-Krenn, 2012). *B*. *longum* subsp. *infantis* is able to metabolize a broad range of HMOs including those that are decorated with fucose and sialic acid (Sela et al., 2008; Zabel et al., 2019). This is due to *B*. *longum* subsp. *infantis* possessing a broad range of ABC transporters specialized to import HMOs which are then further processed by intracellular enzymes (Sela et al., 2008; Wong et al., 2020). In contrast, the ability of *B*. *longum* subsp. *longum* to metabolize HMOs is limited and generally this species can only metabolize LNB and LNT (Galβ1-3GlcNAcβ1-3Galβ1-4Glc), although some *B*. *longum* subsp. *longum* strains can utilize fucosylated HMOs (Garrido et al., 2016). *B*. *longum* subsp. *longum* strains that metabolize HMOs similarly encode ABC transporters for their uptake but can also encode extracellular enzymes to degrade HMOs including an extracellular lacto-*N*-biosidase that cleaves LNT into LNB and lactose (Yamada et al., 2017). *B*. *longum* subsp. *longum* therefore has both the capacity to metabolize HMOs from breast milk in the infant diet and plant-derived oligosaccharides present in the diet of adults. This may be why the *B*. *longum* subsp. *longum* species is found in both the infant and adult gut and is therefore an important bifidobacterial species that is part of the gut microbiota throughout the lifespan of the human host. This knowledge of HMO utilization and plant-derived glycan utilization therefore may be used to encourage an increase in the abundance in bifidobacteria after the weaning and hopefully prevent the decline in bifidobacteria as the human host ages.

## Conclusion and Future Perspectives

The GIT environment is a dynamic, highly competitive and challenging ecological niche for bifidobacteria to colonize. To further complicate matters, the diet of the human host changes as we age, starting from breast milk in infancy to complex plant glycans in adulthood. Therefore, in order to survive the GIT environment bifidobacteria must be able to metabolize complex plant-oligosaccharide carbohydrates and most importantly choose the most metabolically efficient carbohydrate source if it is to compete with other microbial species in the GIT. Bifidobacteria represent a key genus among the gut microbiota and are present in the gut throughout life from infancy, adolescence, adulthood to old age. They are seen as a general indicator of health due to their purported probiotic properties. Therefore, as they decline with human host age it is important to understand how bifidobacterial species adapt and are able to metabolize plant-oligosaccharides more associated in the adult diet. This knowledge may allow the opportunity to increase the abundance of bifidobacteria in the adult and elderly human host potentially benefiting it with the probiotic effects attributed to bifidobacteria.

A key area in carbohydrate metabolism involves the question of how dependent bifidobacteria are on other microbial species (bacterial and/or fungal) to degrade complex insoluble plant glycans into oligosaccharidic substrates? Previously, it has been shown that growth of *Ba*. *cellulosilyticus* on arabinogalactan can support growth of *B*. *breve* by release of galacto-oligosaccharides (Munoz et al., 2020) demonstrating that cross-feeding occurs between species. Further investigations are needed to precisely assess the relationship between bifidobacteria and other species, in particular members of the *Bacteroides* genus. *Bacteroides* spp. are known for their ability to degrade complex plant glycans (Cartmell et al., 2018), and they are called ‘messy eaters’ that extracellularly degrade glycans releasing oligosaccharides for other GIT members, including bifidobacteria, to scavenge (Porter and Martens, 2016). More detailed studies are needed to understand these complex ecological interactions, which may then allow rational strategies to be exploited for the development of novel plant-derived oligosaccharide prebiotics.

Another area related to this research topic includes the role of HCA metabolism by bifidobacteria. Previously, esterases that cleave synthetic HCA substrates have been reported in bifidobacteria (Raimondi et al., 2015; Fritsch et al., 2017; Kelly et al., 2018). The gene encoding the CaeA esterase is located in a locus predicted to be involved in AOS metabolism (Arzamasov et al., 2018; Kelly et al., 2018). Removal of HCAs from plant-derived oligosaccharides is hypothesized to provide substrate access to GHs that might otherwise be sterically hindered by HCA substitutions. However, a lack of commercially available plant-derived oligosaccharide substrates retaining the HCA decorations remains a challenge to ascertain to what extent HCAs affect metabolism of complex plant-derived glycans. Furthermore, do released HCAs, which in *B*. *longum* subsp. *longum* happens intracellularly, provide any benefit to bifidobacteria? In other heterofermentative bacteria HCAs has been shown to act as external electron acceptors and their presence in growth media results in higher intracellular ATP levels (Filannino et al., 2014). HCAs also inhibit growth of certain gut pathogens, such as *Clostridium perfringens* (Lee et al., 2006), presumably due to membrane damage. However, how sensitive bifidobacteria are to the effects of HCAs is currently not studied.

Finally, different plant-oligosaccharides derived from hemi-celluloses and pectin have highly complex structures, yet in cases contain identical monomeric/oligomeric components and glycosidic linkages. Additionally, bifidobacterial genomes often contain multiple loci in different locations across the genome dedicated to the metabolism of dietary carbohydrates. It is likely that if bifidobacteria are provided with a buffet of plant-derived oligosaccharides to metabolize in the gut they must choose the most energetically favorable carbon source as they are competing for resources with other members of the microbiota. In future, more understanding of the bifidobacterial transcriptional regulation of plant derived oligosaccharides is needed and potentially this knowledge could lead to better understanding of the prebiotic plant-oligosaccharides preferentially utilized by bifidobacteria. This, however, requires that plant oligosaccharides are purified to a high quality, that the detailed structural (DP, covalent linkages and sidechain substitutions) information of these oligosaccharides is known and that sufficient amounts of oligosaccharides are purified to allow growth and transcriptional analyses. Currently, plant oligosaccharides are not widely available in large amounts at a reasonable costs, while characterizing oligosaccharides requires specialist techniques and expensive equipment such as mass-spectrometry, HPLC, HPAEC-PAD and NMR. Furthermore, following the acquisition of this information, animal models would need to be employed to assess the prebiotic/bifidogenic potential of a given oligosaccharide. In conclusion, the ability of bifidobacteria to utilize a variety of plant-derived oligosaccharides is an important characteristic of specific members of this genus to colonize and survive in the adult gut. Novel plant-glycan based prebiotics specific for bifidobacteria could be developed in the future, though this will require further research to fully understand plant-derived poly/oligosaccharide metabolic capabilities exerted by bifidobacteria.

## Author Contributions

SK, JM-M, and DS wrote and edited the manuscript. All authors contributed to the article and approved the submitted version.

## Conflict of Interest

The authors declare that the research was conducted in the absence of any commercial or financial relationships that could be construed as a potential conflict of interest.
